# Identification of potential genetic Loci and polygenic risk model for Budd-Chiari syndrome in Chinese population

**DOI:** 10.1016/j.isci.2023.107287

**Published:** 2023-07-11

**Authors:** Xiaojun Hu, Xiaosen Jiang, Jia Li, Ni Zhao, Hairun Gan, Xinyan Hu, Luting Li, Xingtao Liu, Hong Shan, Yong Bai, Pengfei Pang

**Affiliations:** 1 Center for Interventional Medicine, Fifth Affiliated Hospital of Sun Yat-sen University, Zhuhai, China; 2 BGI-Shenzhen, Shenzhen, China; 3 College of Life Sciences, University of the Chinese Academy of Sciences, Beijing, China; 4 BGI Genomics, BGI-Shenzhen, Shenzhen, China; 5 Hebei Industrial Technology Research Institute of Genomics in Maternal & Child Health, Shijiazhuang BGI Genomics Co., Ltd, Shijiazhuang, China; 6 Changfeng Hospital of Jinjiang District, Chengdu, China; 7 Guangdong Provincial Key Laboratory of Biomedical Imaging, Fifth Affiliated Hospital, Sun Yat-sen University, Zhuhai, China; 8 Guangdong Provincial Engineering Research Center of Molecular Imaging, Fifth Affiliated Hospital, Sun Yat-sen University, Zhuhai, China

**Keywords:** Risk factor, Computational molecular modelling, Genetics, Genomics

## Abstract

Budd-Chiari syndrome (BCS) is characterized by hepatic venous outflow obstruction, posing life-threatening risks in severe cases. Reported risk factors include inherited and acquired hypercoagulable states or other predisposing factors. However, many patients have no identifiable etiology, and causes of BCS differ between the West and East. This study recruited 500 BCS patients and 696 normal individuals for whole-exome sequencing and developed a polygenic risk scoring (PRS) model using PLINK, LASSOSUM, BLUP, and BayesA methods. Risk factors for venous thromboembolism and vascular malformations were also assessed for BCS risk prediction. Ultimately, we discovered potential BCS risk mutations, such as rs1042331, and the optimal BayesA-generated PRS model presented an AUC >0.9 in the external replication cohort. This model provides particular insights into genetic risk differences between China and the West and suggests shared genetic risks among BCS, venous thromboembolism, and vascular malformations, offering different perspectives on BCS pathogenesis.

## Highlights

• Exome sequencing found Budd-Chiari syndrome risk mutations, e.g. rs1042331
• A polygenic risk score model for BCS was built using multiple methods, with AUC >0.9
• Genetic risk overlaps were estimated for BCS, VTE, and vascular malformations
• East-West differences in BCS pathogenesis found, e.g. JAK2 gene mutations

## Graphical abstract

**Figure:**
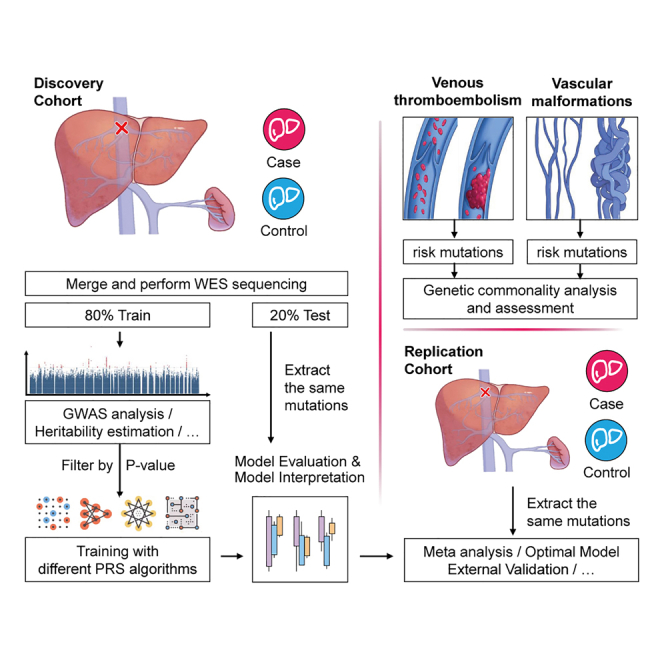

## Introduction

Generalized Budd-Chiari syndrome (BCS) refers to a group of clinical symptoms that involve portal vein thrombosis (PVT) and/or inferior vena cava (IVC) thrombosis.^1^ BCS occurs due to any obstruction in the hepatic venous outflow that is located at any point from the liver to heart^2^ and, in severe cases, may potentially be life-threatening.^1^^,^^3^ BCS is a rare disease in Western countries. However, it has a higher incidence and affects a larger patient population in Eastern countries, such as China.^4^ More than 20,000 BCS cases have been identified in China over the past 30 years.^3^^,^^4^ With advances in diagnostic technologies, the understanding of the etiology and pathology of BCS is constantly improving. Familial clustering of BCS has been described in previous reports.^5^ Gene mutations play a critical role in BCS development. Recent studies have reported that the risk factors for BCS include Leiden mutation of Factor V, prothrombin G20210A mutation, inherited protein C, protein S, or antithrombin deficiencies, myeloproliferative neoplasms (MPNs) caused by gene mutations of *JAK2V617F,*^6^ *CALR,*^7^ or *MPLW515L,*^8^ and paroxysmal nocturnal hemoglobinuria (PNH).^9^

Nevertheless, there are still controversies and uncertainties regarding the diagnosis of BCS.^10^ Due to ethnicity-related and geographical differences, the pathogenesis of BCS in China seems to be significantly different from that in Western countries. The gene mutations mentioned above are rarely found in Chinese BCS patients, which causes difficulties in implementing genetic screening and diagnosis in China.^11^ The lack of large-scale genome sequencing data obtained from Chinese BCS patients has hindered further understanding of the pathogenesis of BCS. In this study, we recruited a large-scale cohort of samples from BCS patients and used the polygenic risk scoring method to construct a BCS risk model. A polygenic risk score (PRS) represents an aggregation of all of the genetic information of an individual, weighted by genetic variants, and provides a simplified score for risk prediction.^12^ PRSs have been widely used in predictions of complex phenotypes and diseases, such as height,^13^ BMI,^14^ Alzheimer’s disease,^15^ and coronary heart disease.^16^ Based on the weighting information for mutation sites in the PRS model, we can speculate about the potential pathogenic mechanisms of complex diseases and provide guidance for prevention and intervention.^17^ We evaluate the application effects of various PRS methods in BCS and further discuss genetic pathogenicity and potential application for BCS risk prediction.

## Results

### Results of whole-exome sequencing and quality control analysis

In the BCS sample group (n = 500) and the healthy sample group (n = 696), more than 95% of the samples showed sequence coverage of at least 30×. The average sequencing depths of the targeted region were 129.44× (range: 71.37–247.92) in the BCS group and 122.08× (range: 79.15–206.84) in the healthy control group (Table S1). After read alignment and the removal of duplicated reads, joint variant calling was performed using GATK HaplotypeCaller,^18^ and 673,764 variants were detected. After the application of VQSR,^18^ VCFTools,^19^ and PLINK^20^ for quality control, a total of 1171 samples (case = 483, control = 688) and 23289 high-confidence variants were retained for the following analysis.

### Identification of potential genetic risk loci through genome-wide association analysis

**Figure 1:**
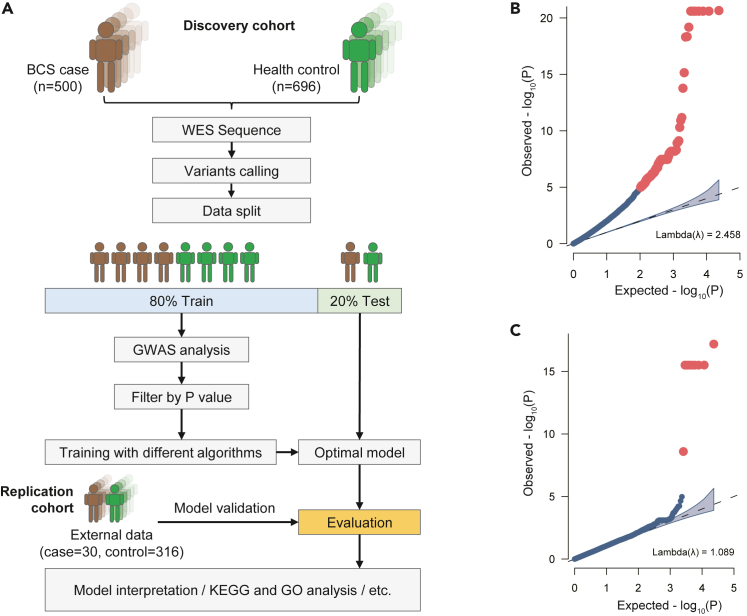
PRS analysis of Budd-Chiari syndrome (A) A total of 1196 samples (case = 500, control = 696) were used for model construction, among which 80% were used for training and 20% were used for testing. The PRS analysis process followed the published PRS guidelines. For the constructed model, 346 external independent samples (case = 30, control = 316) were used for evaluation. For the model results, weight information is extracted for downstream analysis, such as model interpretation and GO and KEGG enrichment analysis. (B) QQ plot of BCS before covariates were added. (C) QQ plot of BCS after covariates were added.

**Figure 2:**
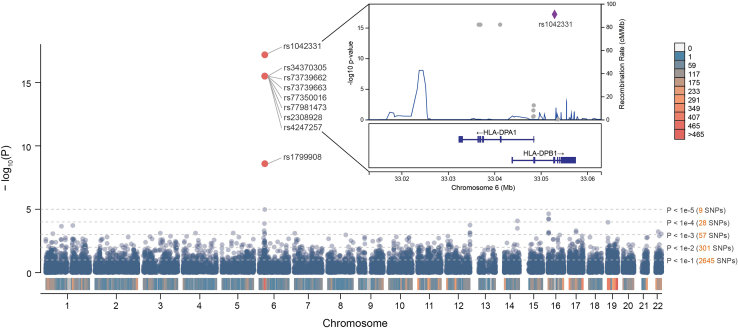
Genome-wide association analysis results for Budd-Chiari syndrome Eight SNPs (rs1042331, rs34370305, rs73739662, rs73739663, rs77350016, rs77981473, rs2308928, rs4247257) were significantly related to BCS and met the condition for whole-genome significance (p value< 5e-8). These sites are located in the 6p21-p32 zone. rs1042331 is located in the *HLA-DPB1* gene. The other 7 SNPs are located within 20 kb upstream of rs1042331 and are located in the *HLA-DPA1* gene. A conditional analysis based on rs1042331 can be viewed in Figure S1.

After performing strict quality control, 23289 high-confidence variants were used for association analysis with the risk of BCS based on the training data (case = 434, control = 619) under an additive model using logistic regression analysis (Figure 1A). Sex, age and the top 10 principal components (PCs) were used as covariates to adjust the GWAS results. Before the covariates were added, there was significant genomic inflation (e.g., λ = 2.46, Figure 1B). After adjustment, the level of genomic inflation, which was mainly due to the significant age differences between the BCS case group and healthy control group (p value = 1.23e-46, Table S2), was controlled (e.g., λ = 1.09, Figure 1C). The results of the GWAS of all 23289 SNPs with the risk of BCS are presented in a Manhattan plot (Figure 2). A total of 8 SNPs were strongly correlated with BCS, including rs1042331 (p = 6.53e-18; OR = 0.28), rs34370305, rs73739662, rs73739663, rs77350016, rs77981473, rs2308928, and rs4247257 (p = 3.12e-16; OR = 0.28), which met the genome-wide significance requirement.^21^ These sites are located in the 6p21-p32 region. We also utilized the genome-wide complex trait analysis (GCTA)^22^ tool to estimate heritability by calculating a genetic relationship matrix (GRM) based on variant data.^23^ The final estimated heritability was 0.58 ± 0.10. Due to factors such as the number of SNPs and nonadditive relationships, this result is likely an underestimate and only reflects the lower limit of heritability for BCS.

To provide a more intuitive visualization of the GWAS results for the most significant SNP site, rs1042331, we employed LocusZoom^24^ to generate a regional association plot. LocusZoom facilitates the visualization of the degree of association between variations near a specific gene locus and the phenotype as well as gene annotation information in the vicinity of the gene locus. We observed that rs1042331 is located within the *HLA-DPB1* gene. The other seven SNPs are situated within 20 kb upstream of rs1042331 and are highly correlated with *HLA-DPA1* (Figure 2). *HLA-DPB* and *HLA-DPA* together form HLA class II molecules anchored on the cell membrane, participate in immune responses, and exhibit significant associations with BCS. We conducted a conditional analysis using the PLINK tool with the mutation site rs1042331 as a covariate. The analysis revealed that the associations of the other seven SNPs with the disease were no longer genome-wide significant (Figure S1), indicating the presence of a unique HLA signal in this region, rather than multiple independent features. Additionally, GWAS of the replication cohort and a meta-analysis based on the two cohorts were performed, and the results are available in Figure S2. For rs1042331, the reference allele was C, and the mutant allele was T. The odds ratios (ORs) for the discovery cohort, replication cohort, and meta-analysis result were 3.62 (2.7–4.86), 7.82 (2.78–22.0), and 3.84 (2.89–5.08), respectively, indicating that the mutant allele (T) was the risk allele.

### Comparison of BCS risk prediction models

**Figure 3:**
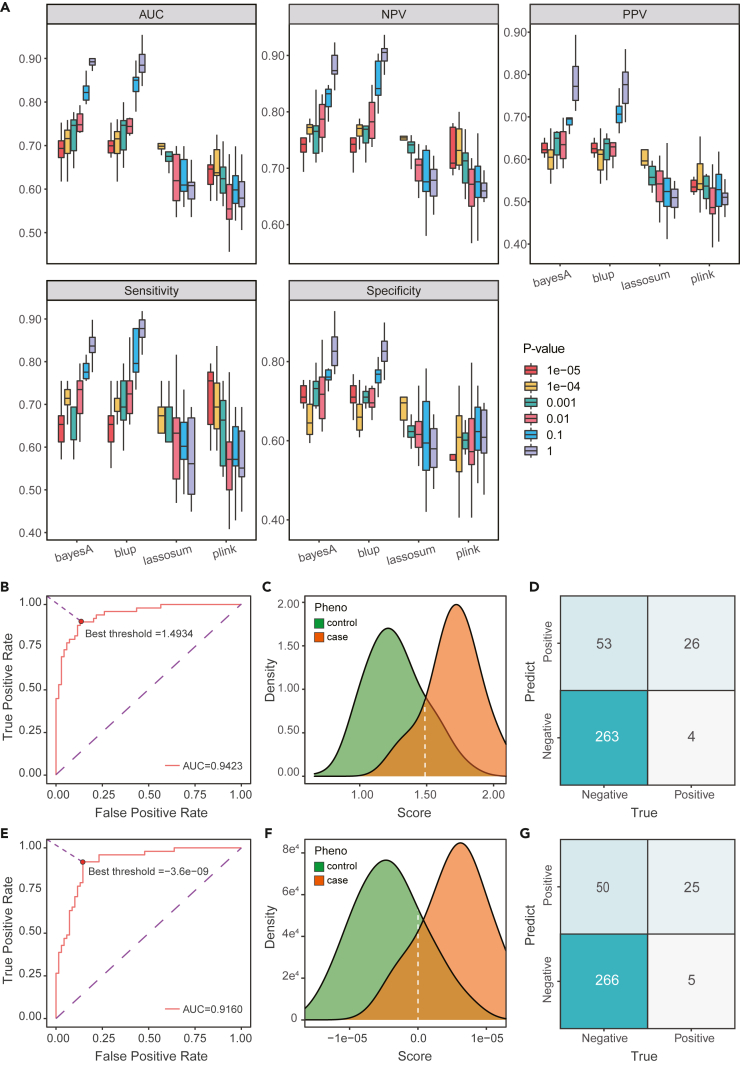
PRS model evaluation analysis (A) The evaluation results for each model were obtained using different algorithms and different p value conditions, with each condition repeated 10 times. (B) The AUC result of the BayesA model was obtained in the discovery cohort internal test set. (C) The distribution of sample scores of the BayesA model was observed in the replication cohort, with the optimal threshold in (B) indicated by the white line. (D) The confusion matrix result for the BayesA model was obtained in the replication cohort using the optimal threshold in (B). (E) The AUC result for the BLUP model was obtained in the discovery cohort internal test set. (F) The confusion matrix result for the BLUP model was obtained in the replication cohort, with the optimal threshold in (E) indicated by the white line. (G) The confusion matrix result for the BLUP model was obtained in the replication cohort using the optimal threshold in (E).

Based on the GWAS results obtained from the training data, different p values (e.g., no filter, ≤10^−1^, ≤10^−2^, ≤10^−3^, ≤10^−4^ and ≤10^−5^) were defined for screening, and the corresponding SNP sets were obtained. Combining these data with the phenotypic data, we used different algorithm models for training using the training dataset and tested them using the test set and replication cohort to understand the model’s ability to assess BCS risk. The best results were obtained from the BayesA^25^ algorithm without SNP filtering (discovery cohort, AUC = 0.94, R^2^ = 0.52; replication cohort, AUC = 0.92, R^2^ = 0.24) (Table 1, Figure 3B), followed by the BLUP^22^ algorithm (discovery cohort, AUC = 0.92, R^2^ = 0.49; replication cohort, AUC = 0.91, R^2^ = 0.21) (Table 1, Figure 3E), LASSOSUM^26^ (discovery cohort, AUC = 0.61, R^2^ = 0.04; replication cohort, AUC = 0.53, R^2^ = 0.001), and PLINK (discovery cohort, AUC = 0.62, R^2^ = 0.04; replication cohort, AUC = 0.53, R^2^ = 0.003) (Table 1). For the BayesA model, we found that as p value filtering became more stringent, the AUC value decreased from 0.94 to 0.69, but the model still maintained better performance compared to other models. To further evaluate the stability of the model, we performed 10-fold cross-validation. We conducted 10 repeated experiments for each model with different p values and plotted boxplots based on different indicators (Figure 3A). The best mean AUC value was 0.90, which was obtained from the BayesA model without p value filtering. The corresponding negative predictive value (NPV) was 0.83–0.92 (mean = 0.88), positive predictive value (PPV) was 0.71–0.89 (mean = 0.78), sensitivity was 0.78–0.90 (mean = 0.84), and specificity was 0.77–0.93 (mean = 0.83). The performance of the BLUP model was similar to that of BayesA. When p values were not filtered, the mean AUC value was 0.89. The corresponding NPVs were 0.81–0.94 (mean = 0.89), PPVs were 0.69–0.86 (mean = 0.77), sensitivities were 0.73–0.92 (mean = 0.86), and specificities were 0.71–0.90 (mean = 0.82). In contrast, LASSOSUM and PLINK were not effective in predicting the risk of BCS. Performance under different p value conditions was low, and model performance gradually decreased with an increasing amount of SNP data.

**Table 1:** Comparison of prediction results of different algorithms in the discovery cohort test set and replication cohort under screening with different p values

p value	Number	Algorithm	Discovery cohort				Replication cohort			
			R^2^	AUC	Odds ratio (Top 50% vs. bottom 50%) (95% CI)	p value (OR)	R^2^	AUC	Odds ratio (Top 50% vs. bottom 50%) (95% CI)	p value (OR)
No filter	23289	**BayesA**	**0.52**	**0.9423**	44.2 (13.59–143.68)	3.01e-10	**0.24**	**0.9238**	34.64 (4.66–257.42)	5.32e-04
		**BLUP**	**0.49**	**0.9160**	6.27 (2.80–14.04)	1.00e-05	**0.21**	**0.9056**	1.98 (1.03–3.79)	0.04027
		LASSOSUM	0.04	0.6075	1.88 (0.90–3.96)	0.09419	0.001	0.5287	0.86 (0.41–1.83)	0.70259
		PLINK	0.04	0.6193	1.76 (0.84–3.70)	0.13526	0.003	0.5301	0.86(0.41–1.83)	0.70259
≤0.1	2645	BayesA	0.36	0.8907	14.52 (5.74–36.76)	1.64e-08	0.16	0.8553	16.51 (3.87–70.49)	1.53e-04
		BLUP	0.43	0.8923	5.72 (2.58–12.68)	2.00e-05	0.16	0.8484	1.74 (0.9–3.37)	0.10270
		LASSOSUM	0.10	0.6758	2.52 (1.19–5.36)	0.01617	0.02	0.6164	1.56 (0.73–3.34)	0.25467
		PLINK	0.11	0.6983	2.65 (1.26–5.59)	0.01044	0.02	0.6290	1.14 (0.56–2.32)	0.71809
≤0.01	301	BayesA	0.37	0.8560	9.56 (4.01–22.76)	3.44e-07	0.16	0.8713	10.48 (3.12–35.25)	1.47e-04
		BLUP	0.36	0.8536	4.11 (1.91–8.84)	0.00031	0.16	0.8741	1.74 (0.9–3.37)	0.10270
		LASSOSUM	0.09	0.6770	2.93 (1.37–6.27)	0.00568	0.04	0.7022	3.64 (1.52–8.72)	0.00381
		PLINK	0.10	0.6803	2.31 (1.10–4.86)	0.02720	0.03	0.6765	1.5 (0.77–2.96)	0.23635
≤1e-3	57	BayesA	0.19	0.7501	3.99 (1.83–8.71)	0.00052	0.08	0.8009	16.51 (3.87–70.49)	1.53e-04
		BLUP	0.19	0.7518	2.65 (1.26–5.59)	0.01044	0.09	0.8052	1.89 (0.98–3.65)	0.05559
		LASSOSUM	0.10	0.6800	2.93 (1.37–6.27)	0.00568	0.06	0.7581	10.48 (3.12–35.25)	0.00015
		PLINK	0.06	0.6205	1.88 (0.90–3.96)	0.09419	0.04	0.7232	1.74 (0.9–3.37)	0.10270
≤1e-4	28	BayesA	0.11	0.6980	3.41 (1.58–7.37)	0.00180	0.06	0.7741	16.51 (3.87–70.49)	1.53e-04
		BLUP	0.11	0.6989	2.48 (1.18–5.21)	0.01709	0.06	0.7769	1.89 (0.98–3.65)	0.05559
		PLINK	0.05	0.6350	2.31 (1.10–4.86)	0.02723	0.05	0.7310	1.58 (0.81–3.09)	0.18126
≤1e-5	9	BayesA	0.11	0.6920	3.41 (1.58–7.37)	0.00180	0.04	0.7331	10.48 (3.12–35.25)	1.47e-04
		BLUP	0.11	0.6920	2.48 (1.18–5.21)	0.01709	0.04	0.7331	1.81 (0.94–3.5)	0.07597
		PLINK	0.06	0.6281	2.31 (1.10–4.86)	0.02723	0.04	0.6944	1.58 (0.81–3.09)	0.18126

Model performance of interest in model interpretation is bolded.

### External verification of the BayesA and BLUP models

We found that the BayesA and BLUP models had better BCS risk prediction effects by using model comparisons. To further verify the performance of these two models, we used 346 individual samples, representing 30 BCS samples and 316 controls, for external verification (Figure 1A). Under the optimal conditions (e.g., without p value screening, SNPs = 23286), we extracted the genotype data of these SNPs from the samples and combined these data with the weight information of the SNPs in the model to calculate the BCS risk score of each sample. The score density distributions of the BCS and healthy individuals for the two models are shown in Figures 3C and 3F. The obtained confusion matrix based on the optimal threshold is shown in Figures 3D and 3G. For the BayesA model, AUC = 0.92, NPV = 0.99, PPV = 0.33, sensitivity = 0.87, and specificity = 0.83; for the BLUP model, AUC = 0.91, NPV = 0.98, PPV = 0.33, sensitivity = 0.83, and specificity = 0.84. The BayesA and BLUP models had high sensitivity and specificity for BCS risk prediction. However, due to the low incidence of BCS, the real sample dataset was not balanced, which led to more false-positive samples in the prediction process.

### Model interpretation

**Figure 4:**
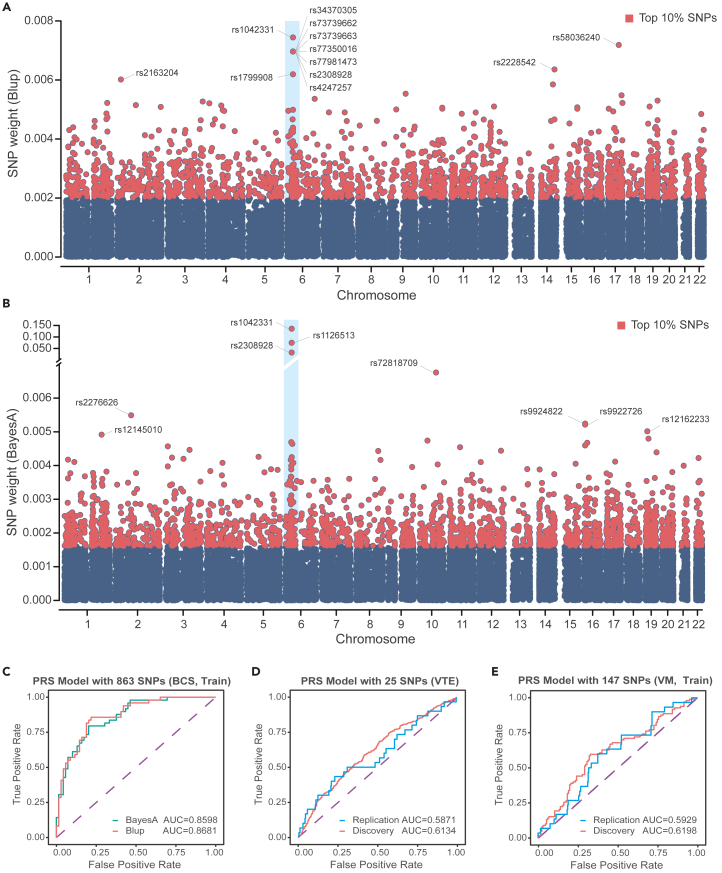
PRS model interpretation and genetic commonality analysis (A and B) Weight distribution diagram of the BLUP and BayesA optimal model; the weight is the absolute value of the SNP effect value in the model. (C) AUC result for the BLUP and BayesA model constructed based on the intersection of the top 10% of SNPs in (A) and (B). (D) AUC result for the VTE 25 SNP PRS model. (E) AUC result for the VM 147 SNP PRS model.

We extracted the weights of all SNPs in the BayesA and BLUP models (without p value screening, SNPs = 23286), calculated the absolute values of the weights, and plotted the weight distributions (BLUP model, Figure 4A; BayesA model, Figure 4B). There were a large number of low-weight SNPs in the two models. The SNP with the greatest weight, rs1042331, was the same in both models and was also the most significant in the GWAS (p = 6.53e-18; OR = 0.28). Some of the top-ranked SNPs were labeled (Figures 4A and 4B). We found that these SNPs had low degrees of overlap between the two models, and only rs1042331 and rs2308928 were identified. This may have been caused by the differences in the algorithms. For the complex disease BCS, an explanation that uses a single reported mutation is generally insufficient. For example, the Factor V Leiden (*F5*) mutation only explains 17%–32% of cases the total BCS population.^27^ Here, we focused on a group of variant sites with high weights in two models. We extracted those SNPs with the top 10% of absolute weights from both models and obtained the intersection. A total of 863 SNPs were obtained, and we speculated that these 863 SNPs contributed more to BCS risk assessments. To further confirm our speculation, we reused BayesA and BLUP for training against these 863 SNPs and validated them by using the test set. BayesA had an AUC of 0.86, while BLUP had an AUC of 0.87 (Figure 4C), and both still maintained good performance.

In addition, we mapped these SNPs to genes and obtained a total of 700 genes. We performed a comprehensive gene set enrichment analysis of these genes using Enrichr,^28^ which included Gene Ontology (GO) terms, KEGG pathways, and other gene sets. We extracted the results related to thrombosis (Table 2), which showed that after multiple corrections of the p values, several pathways were identified as significant (Adjusted p value <0.05), including the composition of lipid Particles (WP3601), metabolic pathways of LDL, HDL, and TG, including disease (WP4522), coexpression of *JAK2* human kinase *ARCHS4*, and metabolite levels (measured by lipoproteins). We also found that these genes were enriched in the set of downstream genes regulated by *JAK2*. Protein functional domains of these genes such as VWFA, FN III, and EGF-like, were also enriched. Enrichr provided a correlation network based on these gene sets (Figure S3).

**Table 2:** The results of enrichment analysis of some gene sets that may be related to BCS are displayed

Gene Set	Term	Overlap	p value	Adjusted p value	Odds Ratio	Combined Score	Related reports (PMID)
WikiPathway 2021 Human	**Composition of Lipid Particles WP3601**	**4/9**	**1.63E-04**	**0.032**	**22.17**	**193.44**	**19683659**
	**Metabolic pathway of LDL, HDL and TG, including diseases WP4522**	**5/16**	**1.64E-04**	**0.032**	**12.61**	**109.95**	**16848878**
	Purinergic signaling WP4900	6/33	8.88E-04	0.115	6.17	43.36	21586363
Reactome 2016	Chylomicron-mediated lipid transport Homo sapiens R-HSA-174800	5/17	2.26E-04	0.106	11.56	97.09	33483737
	FGFR1 mutant receptor activation Homo sapiens R-HSA-1839124	6/31	0.000627	0.124	6.66	49.16	15863718
Panther 2016	Endothelin signaling pathway Homo sapiens P00019	9/75	0.001195	0.086	3.79	25.54	31747868,
NCI-Nature 2016	Regulation of RAC1 activity Homo sapiens 351aacd6-6195-11e5-8ac5-06603eb7f303	6/38	0.001909	0.326	5.2	32.59	21757621
	IL23-mediated signaling events Homo sapiens b71d0ffd-6193-11e5-8ac5-06603eb7f303	5/37	0.008928	0.606	4.33	20.43	28951241
Elsevier Pathway	Hippo/YAP1 Signaling	6/31	6.27E-04	0.679	6.66	49.16	31024911
GO Biological Process 2021	peptidyl-lysine deacetylation (GO:0034983)	4/8	9.31E-05	0.193	27.72	257.34	22767499
ARCHS11 Kinases Coexp	**JAK2 human kinase ARCHS4 coexpression**	**23/299**	**3.71E-04**	**0.02**	**2.34**	**18.49**	**32850227**
Kinase Perturbations from GEO down	JAK2 active mutant 72 GSE21842	21/300	0.002131	0.607	2.1	12.96	32850227
ClinVar	familial hypercholesterolemias	3/8	0.002095	0.056	16.6	102.44	19004443
	hyperlipoproteinemia	3/8	0.002095	0.056	16.6	102.44	31808903
GWAS Catalog 2019	**Metabolite levels (lipoprotein measures)**	**7/27**	**3.01E-05**	**0.027**	**9.73**	**101.38**	**21145806**
	Cardiovascular disease risk factors	5/20	5.18E-04	0.116	9.24	69.97	–
Human Gene Atlas	Fetal liver	12/126	0.001573	0.127	2.93	18.94	–
Pfam InterPro Domains	**VWF A**	**9/49**	**4.38E-05**	**0.008**	**6.27**	**62.93**	**17392288**
	RhoGEF	8/63	0.001532	0.076	4.04	26.21	24081657
	FN III	13/151	0.002541	0.102	2.62	15.69	20116835
	EGF like	11/133	0.0071	0.157	2.5	12.41	11848442
	PH	16/235	0.008808	0.157	2.03	9.64	32548550
	Laminin G 2	5/37	0.008928	0.157	4.33	20.43	24741378

Pathways that are strictly significant, with multiple-adjusted p values <0.05, are highlighted in bold.

### Results of rare variant aggregate analysis

We detected 114,204 rare variants (minor allele frequency <0.05) in the BCS group in the discovery cohort and performed the SNP-set (Sequence) Kernel Association Test (SKAT) analysis^29^ to characterize the association of genes with BCS. The genes *PRRC2A*, *CSNK1E*, and *KDM6B* showed significant associations with BCS based on SKAT analysis (p < 1.43e-06 for all cases). Known BC risk genes and genes in the *JAK2* subnetwork (*JAK2* human kinase *ARCHS4* coexpression) were not significantly enriched for rare variants in the SKAT analysis (p < 1.43e-06 for all cases; Table S3).

### Genetic commonality analysis

According to the scoring results of the venous thromboembolism (VTE) 25 SNP model and the vascular malformations (VM) 147 SNP model, combined with the real labels of the samples, we drew ROC curves (Figures 4D and 4E). For BCS risk prediction, the VTE 25 SNP model had an AUC of 0.61 and R2 of 0.04 in the discovery cohort and an AUC of 0.59 and R2 of 0.01 in the replication cohort. The VM 147 SNP model had an AUC of 0.62 and R2 of 0.03 in the discovery cohort and an AUC of 0.59 and R2 of 0.01 in the replication cohort. We further sorted the scores based on threshold ratios of the top 5%, 10%, 20%, and 50%, stratified the samples, and calculated odds ratios (Table 3). The odds ratios of the VTE 25 SNP model ranged from 3.25 (95% CI, 0.70–0.79) to 2.19 (1.43–3.55) in the discovery cohort and from 1.34 (0.29–6.12) to 1.82 (0.84–3.94) in the replication cohort. The odds ratios of the VM 147 SNP model ranged from 3.31 (0.70–15.69) to 2.41 (1.41–4.10) in the discovery cohort and from 4.66 (1.54–14.13) to 1.16 (0.55–2.45) in the replication cohort. The VTE 25 SNP model was a VTE risk prediction model. The scores reflected the disease risk for VTE. In association with BCS, it was found that the higher the score of each patient, the higher the risk of developing BCS. In the VM 147 SNP model, the mutation sites of the VM-related gene regions were used to construct the BCS risk prediction model. Based on the odds ratios, a higher risk score is associated with a greater risk of BCS in the general population.

**Table 3:** Evaluation of the predictive effect of the PRS risk prediction model for VM and VTE diseases on BCS to assess the common genetic risk among the diseases

PRS Model	Discovery cohort				Replication cohort					
	Risk	Reference	Odds Ratio (95% CI)	p value (OR)	R^2^	AUC	Odds Ratio (95% CI)	p value (OR)	R^2^	AUC
PRS Model with 25 SNPs (VTE)	Top 5	Remaining 95	3.25 (0.70–15.13)	0.13247	0.04	0.6134	1.34 (0.29–6.12)	0.70643	0.0079	0.5929
	Top 10	Remaining 90	1.72 (0.70–4.21)	0.23770			0.99 (0.28–3.43)	0.98247		
	Top 20	Remaining 80	2.05 (1.07–3.94)	0.03066			1.49 (0.63–3.5)	0.36114		
	Top 50	Remaining 50	2.19 (1.43–3.35)	0.00030			1.82 (0.84–3.94)	0.13079		
PRS Model with 147 SNPs (VM, Self-training)	Top 5	Remaining 95	3.31 (0.70–15.69)	0.13098	0.03	0.6198	4.66 (1.54–14.13)	0.00652	0.0114	0.5871
	Top 10	Remaining 90	1.69 (0.67–4.27)	0.27034			2.47 (0.94–6.54)	0.06795		
	Top 20	Remaining 80	2.11 (1.05–4.26)	0.03649			2.13 (0.95–4.79)	0.06657		
	Top 50	Remaining 50	2.41 (1.41–4.10)	0.00124			1.16 (0.55–2.45)	0.70259		

The samples were sorted based on the score of the PRS model and grouped according to the proportions of 5%, 10%, 20%, and 50%, respectively. The OR was then calculated to evaluate the predictive effect of the PRS score on BCS.

## Discussion

The GWAS results indicated that rs1042331, rs34370305, rs73739662, rs73739663, rs77350016, rs77981473, rs2308928, and rs4247257 were significantly related to BCS; these SNPS are located in *HLA-DPB1* and *HLA-DPA1*, and this region can be regarded as an independent signal region. A previous report showed potential correlations between *HLA-DPB1* and *HLA-DPA1* and thrombosis and ANCA-associated vasculitide (AAV)-associated thrombus formation.^30^ Our results suggested that *HLA-DPB1* and *HLA-DPA1* were related to the formation of BCS.

We used different methods to construct the PRS model. The results showed that the BayesA and BLUP methods were more effective and applicable to cases with more SNPs as features. In the replication cohort, the AUC was 0.92 for BayesA and 0.91 for BLUP (no p value screening, SNPs = 23286). We found that the AUCs, NPVs, specificities and sensitivities of these models were satisfactory, but the PPVs were low in both the BayesA (0.78) and BLUP (0.77) models, and the false-positive rates were high. This may be due to the low incidence of BCS and sample imbalance in the real environment. Nevertheless, we can still use the BCS risk prediction model for large-scale preliminary screening and for further clinical diagnoses or risk prompts for high-risk populations. In view of its high positive detection rate, we can still ensure that BCS patients are diagnosed in time and carry out intervention treatments.

In the interpretation of the BayesA and BLUP PRS models, we found some interesting pathways, and we suspect that these pathways are potentially related to the formation of BCS or thrombi. The gene enrichment pathways corresponding to the top 10% of the absolute weights of the SNPs in the two models are related to lipoproteins, lipid transport metabolism, endothelin, and some protein families related to thrombosis, such as those with VWF A and EGF-like domains. Interestingly, these genes are also enriched in the *JAK2* gene regulatory network. *JAK2* mutations and Factor V Leiden mutations have been reported to be associated with BCS but have not been detected in large numbers in Chinese BCS patients. We quantified the incidence rates of *JAK2V617F* and Factor V Leiden mutations in the normal and BCS groups in all samples (normal = 1012, BCS = 530) as follows: *JAK2V617F* (normal = 0%, BCS = 2.83%) and Factor V Leiden mutation (normal = 0.19%, BCS = 0.10%). Therefore, we speculate that due to population differences, Chinese BCS patients are more likely to have mutations in *JAK2*-related genes that exert the same pathogenic effect as *JAK2* gene mutations. In addition, an analysis of genetic commonality with VTE and VM showed that the risk factors for BCS might overlap with those for VTE and VM. The high-risk VTE and VM groups might also correspond to the high-risk BCS groups. These groups are all reported to show an association with the formation of thrombi. Therefore, we speculate that they may share risk factors related to thrombosis.

### Limitations of the study

In this study, the number of samples was not sufficiently large, which may have caused some genetic risk factors to be undetected. Our sample population consisted only of Chinese people, which may cause the PRS model to be unsatisfactory for Europeans and other populations. The current model can be used only for extensive screening and predicting risks. We may need to further expand the sample size or develop a new PRS algorithm to reduce the probability of false-positives.

## STAR★Methods

### Key resources table

**Table:** 

REAGENT or RESOURCE	SOURCE	IDENTIFIER
**Biological samples**		

Venous blood	Cohort sample in article	N/A

**Critical commercial assays**		

MagPure buffy coat DNA midi KF kit	Magen, Guangzhou, China	https://www.magen-tec.com/
MGIEasy Exome Capture V4 Probe Set	MGI, Shenzhen, China	https://en.mgi-tech.com/products/reagents_info/9/
Segmentase	BGI, Shenzhen, China	http://www.bgitechsolutions.com/sequencing/105
MGIseq-2000 platform	MGI, Shenzhen, China	https://en.mgitech.cn/Uploads/Temp/file/20200115/5e1e68f7779a5.pdf
Whole exome sequencing service	BGI, Shenzhen, China	http://www.bgitechsolutions.com/sequencing/105

**Deposited data**		

Raw analysis data + code	GitHub	https://github.com/jxs1996/Budd-Chiari-syndrom Contains the GWAS analysis results of the discovery cohort and the repeat cohort, the original analysis code for statistical analysis, some supplementary materials, etc.
PRS model of VTE	PGS catalog project	https://www.pgscatalog.org/score/PGS000043/
Raw sequencing data	China National GeneBank DataBase (CNGBdb)	The Discovery Cohort data that support the findings of this study have been deposited into CNGB Sequence Archive (CNSA) of China National GeneBank DataBase (CNGBdb) with accession number CNP0002226.
NHLBI Exome Sequencing Project (ESP6500)	NHLBI Exome Sequencing Project (ESP)	https://evs.gs.washington.edu/EVS/
dbsnp141 databases	NCBI dbSNP Database	https://www.ncbi.nlm.nih.gov/projects/SNP/snp_summary.cgi?view+summary=view+summary&build_id=141
1000 Genomes	1000 Genomes Project	https://www.internationalgenome.org/
Exome Aggregation Consortium (ExAC) and Genome Aggregation Database (gnomAD)	Genome Aggregation Database	https://gnomad.broadinstitute.org/

**Software and algorithms**		

PLINK v2.0 alpha	Christopher Chang,	https://www.cog-genomics.org/plink/2.0/
GATK	Broad Institute	https://gatk.broadinstitute.org/hc/en-us
BWA	Heng Li	https://github.com/lh3/bwa
VCFTools	Richard Durbin	https://vcftools.sourceforge.net/
GCTA	Jian Yang	https://yanglab.westlake.edu.cn/software/gcta/#Overview
LASSOSUM	Pak Chung Sham	https://github.com/tshmak/lassosum
BLUP	Jian Yang	https://yanglab.westlake.edu.cn/software/gcta/#BLUP
KGGseq	Miaoxin Li	http://pmglab.top/kggseq/
bWGR R package BayesA algorithm	Katy M Rainey	https://doi.org/10.1093/bioinformatics/btz794
LocusZoom	Cristen J. Willer	http://locuszoom.org/
R 4.1.0	R CRAN	https://www.r-project.org/
Python	Python Software Foundation	https://www.python.org/

### Resource availability

#### Lead contact

For more information and requests for raw data and codes, please contact Xiaojun Hu (huxiaoj5@mail.sysu.edu.cn).

#### Materials availability

This study did not generate new unique reagents.

### Experimental model and subject details

#### Sample recruitment and ethics statement

In this study, we recruited 500 BCS patients without a known disease cause from the Fifth Affiliated Hospital of Sun Yat-sen University and the Affiliated Hospital of Xuzhou Medical University. We assessed all known causes of BCS, including acquired disorders, inherited disorders, endocrine disorders, and local lesions. Enhanced computed tomography (CT) or magnetic resonance imaging was used to evaluate the specific imaging findings of BCS. Additionally, we enrolled 696 healthy individuals as controls at our hospital based on laboratory testing, imaging evaluation, history taking and physical examination. All healthy participants underwent medical check-ups in our hospital and were confirmed to have no history of BCS. The above samples were used as the discovery cohort as internal data for building the model and evaluating the predictive accuracy of the model. In addition, we recruited an additional 30 BCS patients and 316 healthy individuals as a replication cohort using the same inclusion criteria as described above. These samples were used as independent external data to validate the performance of the model. Written informed consent was obtained from all participants. This study was approved by the Institutional Review Board of the Fifth Affiliated Hospital, Sun Yat-sen University (K05-1) and the Institutional Review Board of BGI (BGI-IRB 21097).

#### Whole-exome sequencing and quality control

Five milliliters of venous blood was taken from each of the participants, and genomic DNA was extracted according to the manufacturer’s standard procedure for the MagPure Buffy Coat DNA Midi KF Kit (Magen, Guangzhou, China). Genomic DNA was fragmented using Segmentase (BGI, Shenzhen, China) to generate small DNA fragments (e.g., 100–500 bp), which were passed over magnetic beads to enrich the fragments that were 280–320 bp long. The ends were filled in, and then an "A" base was added to the 3' end to allow the DNA fragment to be ligated to an adapter bearing a "T" base at the 3' end. The resulting DNA fragments were amplified by ligation-mediated polymerase chain reaction, purified, and then used to create a library. The library was enriched by array hybridization using the MGIEasy Exome Capture V4 Probe Set (MGI, Shenzhen, China), followed by elution and postcapture amplification. The products were then analyzed on an Agilent 2100 Bioanalyzer to estimate the magnitude of enrichment. All amplified libraries were subsequently sent to BGI for circularization and sequencing on the MGIseq-2000 platform (BGI, Shenzhen, China) with an average depth of ∼120X (PE100). For the original sequencing data, we removed unqualified sequences from the primary data using a local dynamic programming algorithm to generate "clean reads".^31^ This included low-quality reads, adapter sequences, and reads containing more than 10% Ns, 50% reads with a quality value of less than 5, and an average quality of less than 10. The “clean reads” were then aligned to the human genome reference (hg19) using Burrows Wheeler Aligner (BWA) software, which was followed by the removal of PCR duplicates with the Picard tool, local indel realignment, base quality score recalibration, and joint variant calling with GATK HaplotypeCaller. We applied the Variant Quality Score Recalibration (VQSR) method to filter out potential low-quality variants with the default datasets and used the parameters recommended by the GATK toolkit.

For the VCF file obtained above, we conducted stringent quality control based on the published PRS guidelines.^32^ VCFTools software was utilized to further remove unqualified variants and samples, and specific parameters were defined as follows^19^: --remove-filtered-all, --remove-filtered-geno-all, --remove-indels, --minGQ 20, --minDP 30, --mac 3, --max-meanDP 100, --max-missing-count 1, --hwe 1e-6, --min-alleles 2, and --max-alleles 2. We then used PLINK software to retain single nucleotide polymorphisms (SNPs) with minor allele frequencies (MAF) > 0.05 that were located on the autosomes and eliminated those individuals with high affinity (--*rel*-cutoff 0.125) and high heterozygosity (e.g., F coefficients that were greater than 3 standard deviations from the mean).^20^

### Method details

#### Preparation of the training and testing sets for the PRS model

To develop and test the PRS model, we followed best practices to ensure unbiased model performance estimates by developing the models using datasets that were distinct from the datasets that were used to test the model performance.^32^ For the discovery cohort, we randomly sampled 90% of the samples from the quality-controlled data as training data and the remaining 10% as testing data. The ratio of case and control samples in the test set and training set was consistent with the dataset before splitting. The replicate cohort was used as an independent dataset for further validation of the model.

#### Genome-wide association study (GWAS) and heritability estimation

GWASs are commonly used to assess the association between a large number of genetic variations (mainly SNPs) and traits or diseases.^33^ In our GWAS analysis, we defined common variants using the minor allele frequency (MAF). Typically, variants with an MAF greater than 1% or 5% are considered common. In this study, we set the MAF threshold at 5%, meaning that only variants with an MAF greater than 5% were considered common. The GWAS was conducted using PLINK software. For the binary BCS variables, we used logistic regression for model fitting, and the SNPs were independent of each other. In addition, we performed principal component analysis (PCA) on the SNP data of the training set and extracted the top 10 principal components (PCs) of all samples as well as their sex and age. These data were used as covariates to remove the potential influence of population stratification and other cryptic correlations on association analyses. In addition, we also used the SNP data after quality control to estimate heritability. The process used the GCTA tool and first calculated a genotype-based GRM. A REML model is then run combining the phenotype data, covariate data and GRM to estimate heritability.

#### PRS estimations with different methods

We used the PLINK, LASSOSUM,^26^ BLUP,^22^ and BayesA^25^ methods to calculate the PRS risk scores, and their descriptions are as follows:

PLINK is a simple additive model that does not require training. This method calculates scores by simply adding the effect values of the SNPs in the GWAS summary data. The effect values are typically reported as log odds ratios (e.g., log(OR)). PLINK does not directly provide a PRS calculation method considering LD, but we use its own parameters --indep-pairwise 50 5 0.2 for LD pruning to remove SNPs with high linkage disequilibrium (LD).

LASSOSUM is a method for calculating LASSO/Elastic Net estimates of a linear regression problem given summary statistics from GWAS and genome-wide meta-analyses, which account for LD via a reference panel.

BLUP is the best linear unbiased prediction. The linear mixed model was y = μ + Xb + e, where y was the response variable, μ was the intercept, X were the input features, b was the regression coefficient, and e was the residual coefficient. Although the BLUP method does not directly account for LD, it can be incorporated by using the Genome Relationship Matrix (GRM). The GRM is a symmetric matrix that measures the genetic similarity between individuals. When calculating the GRM, LD can be taken into account to reduce redundant information among genetic variants.

BayesA was built using the R package bWGR. It assumes that each SNP has an effect and follows a normal distribution and that the effect variance follows a scaled inverted chi-square distribution χ–2(ν, S), where S is the scale parameter and ν is the degree of freedom. BayesA introduces Gibbs sampling into Markov chain Monte Carlo theory (MCMC) to calculate the effect size of SNPs and considers and calculates LD between adjacent markers in a forward Markov model.

All methods were evaluated using different SNP feature sets. The SNPs were filtered using the GWAS association p values at thresholds of 10^−1^, 10^−2^, 10^−3^, 10^−4^ and 10^−5^. These were also tested using the full SNP feature set without any filtering.

#### Model evaluation and interpretation

Using the risk scores that were provided by each method, the receiver operating characteristic (ROC) curve was drawn, and the area under the ROC curve (AUC) value was calculated. The AUC is currently considered to be the standard method for assessing the accuracy of predictive models, with AUC = 1.0 representing perfect performance and 0.50 indicating a random guess. In addition, we calculated the accuracy, sensitivity and other indicators and used these indicators to determine the best prediction method for BCS. Furthermore, we extracted the weight information of the SNPs corresponding to the model and the genes in which the SNPs were located. We then used Enrichr^28^ to perform enrichment analysis of these genes to evaluate their enrichment levels in different gene sets.

#### Associations of rare variants and genes with disease

We defined rare variants as those with a minor allele frequency lower than 5% in the NHLBI Exome Sequencing Project (ESP6500) and dbsnp141 databases as well as among East Asian subjects in the 1000 Genomes, Exome Aggregation Consortium (ExAC) and Genome Aggregation Database (gnomAD) databases. KGGseq^34^ software was used to examine the association between rare variants and BCS. The SNP-set (Sequence) Kernel Association Test (SKAT) was used to examine the association between a set of rare variants and dichotomous phenotypes using kernel machine methods.^29^

#### Relevant disease PRS model construction to predict BCS risk

According to a previous report, we found that some BCS patients had vascular abnormalities,^35^ which are reminiscent of clinically different vascular malformations (VMs) with thrombotic events.^36^ Further research showed that they may be caused by defects in the development of blood vessels that are caused by congenital mutations.^37^ Venous thromboembolism (VTE) has also been found to show the same genetic risk factors as BCS, such as Leiden mutation of Factor V, protein C, and protein S.^38^ To explore their genetic commonalities, we attempted to predict BCS risk based on reported PRS models or genetic risk information for these diseases. By predicting the outcomes, we can assess whether the risk factors for these diseases have risk trends that are consistent for BCS.

We created 2 PRS models. 1) VTE 25 SNP model: We downloaded the PRS model of VTE (pgs000043) from the PGS catalog project (https://www.pgscatalog.org/). It was mainly constructed based on European individuals and contained 297 variants. These variants intersected with our project data, and a total of 25 variants were retained. We extracted the weights of these variants, constructed a new model and used it to score our 1171 samples. 2) VM 147 SNP model: Since no reported PRS model associated with VM was found, we performed an extensive literature search and collected a large number of genes reported to be involved in VM (Table S4). A total of 147 SNPs located in these genes were filtered out in the dataset of this project. Training was performed on our training set (n = 936) by using the BayesA method, and scoring was performed on the test set (n = 235).

### Quantification and statistical analysis

For data quality control, we used a threshold of MAF = 0.05, where mutations greater than 5% were used for PRS model building and mutations below 5% were used for gene-based rare variant clustering analysis. Other quality control conditions can be found in the method details section. For GWAS and heritability estimation, we used sex, age, and the top 10 principal components (PCs) as covariates for correction. The results corresponding to the analysis software and related statistics were extracted and displayed in the final chart. The specific process for constructing the PRS model is shown in Figure 1. It should be noted that the results in Figure 3A are the statistical results of the 10-fold cross-validation of the discovery cohort and were drawn using R. For gene-based rare variant aggregation analysis, we defined the Bonferroni-corrected gene-level exome-wide significance threshold to be p = 0.05/(2 tests × 17,427 genes) = 1.43e-06.

## Data Availability

• Data: The openly available data in this article has been uploaded to the GitHub project (https://github.com/jxs1996/Budd-Chiari-syndrom), and the corresponding data can be accessed and downloaded from the key resources table. • Code: The analysis code is stored in the github project (https://github.com/jxs1996/Budd-Chiari-syndrom). • Any additional information required to reanalyze the data reported in this paper is available from the lead contact upon request.

## Supplemental information

Document S1. Figures S1–S3, Tables S1, S2 and S4

Table S3. SKAT analysis results, related to Figure 2
