# Supplementary material for: Identification of potential genetic Loci and polygenic risk model for Budd-Chiari syndrome in Chinese population
Source: iScience. 2023 Jul 11;26(8):107287. doi: 10.1016/j.isci.2023.107287 (PMC10393737; doi:10.1016/j.isci.2023.107287)
Supplement: Document S1. Figures S1–S3, Tables S1, S2 and S4 [file mmc1.pdf]

iScience, Volume *26*

Supplemental information

Identification of potential genetic Loci and polygenic risk model for Budd-Chiari syndrome in Chinese population

Xiaojun Hu, Xiaosen Jiang, Jia Li, Ni Zhao, Hairun Gan, Xinyan Hu, Luting Li, Xingtao Liu, Hong Shan, Yong Bai, and Pengfei Pang

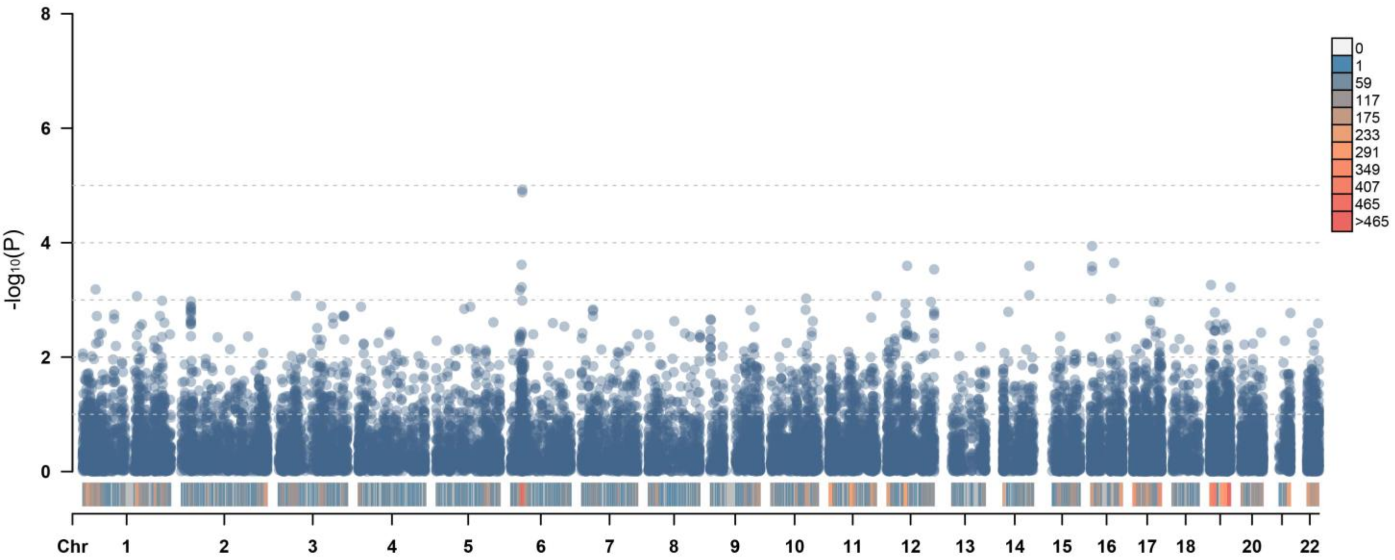

Figure S1. Manhattan plot after conditional analysis based on rs1042331, Related to Figure 2.

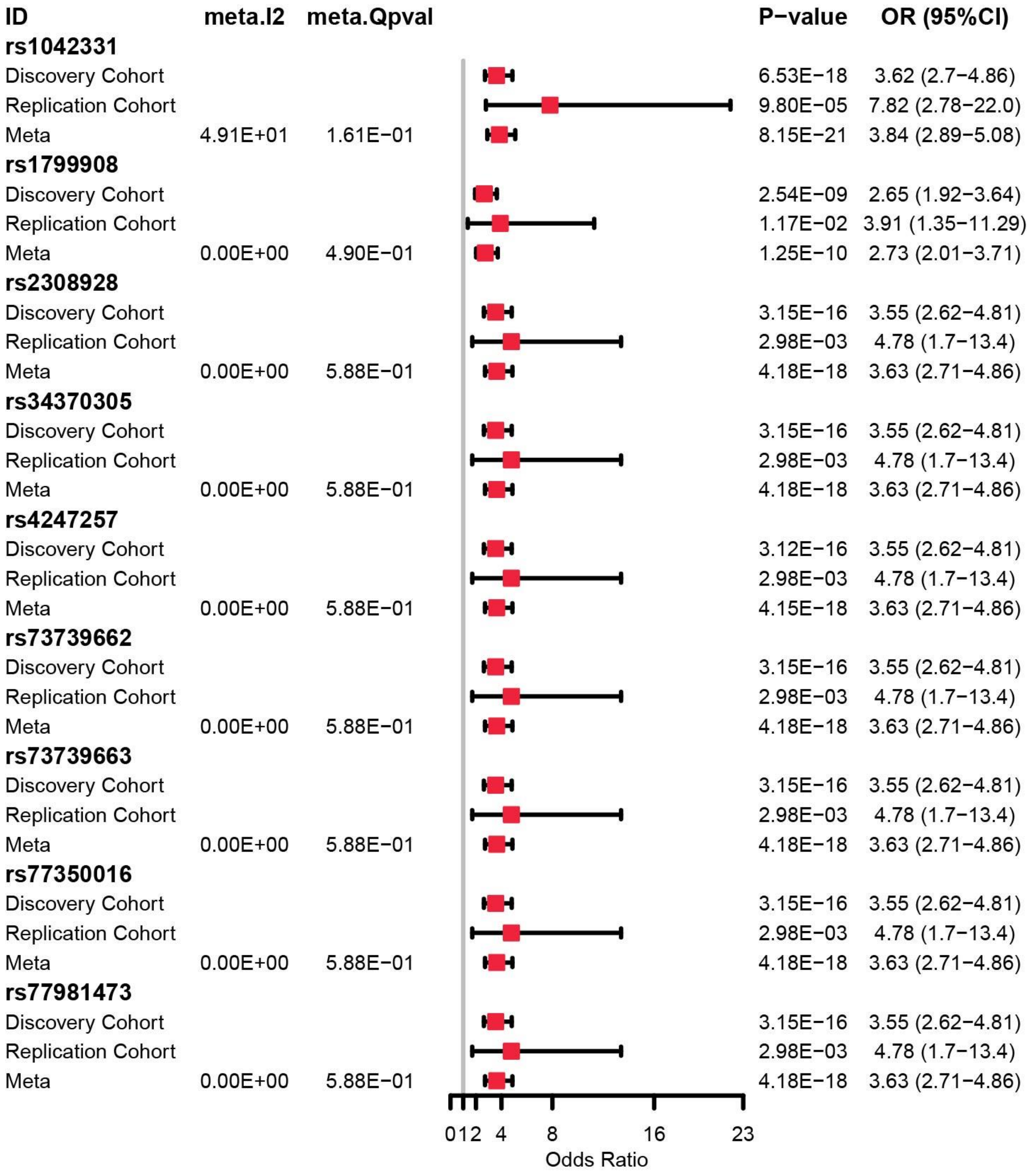

Figure S2. Meta-analysis results based on the discovery cohort and replication cohort, Related to Figure 2.

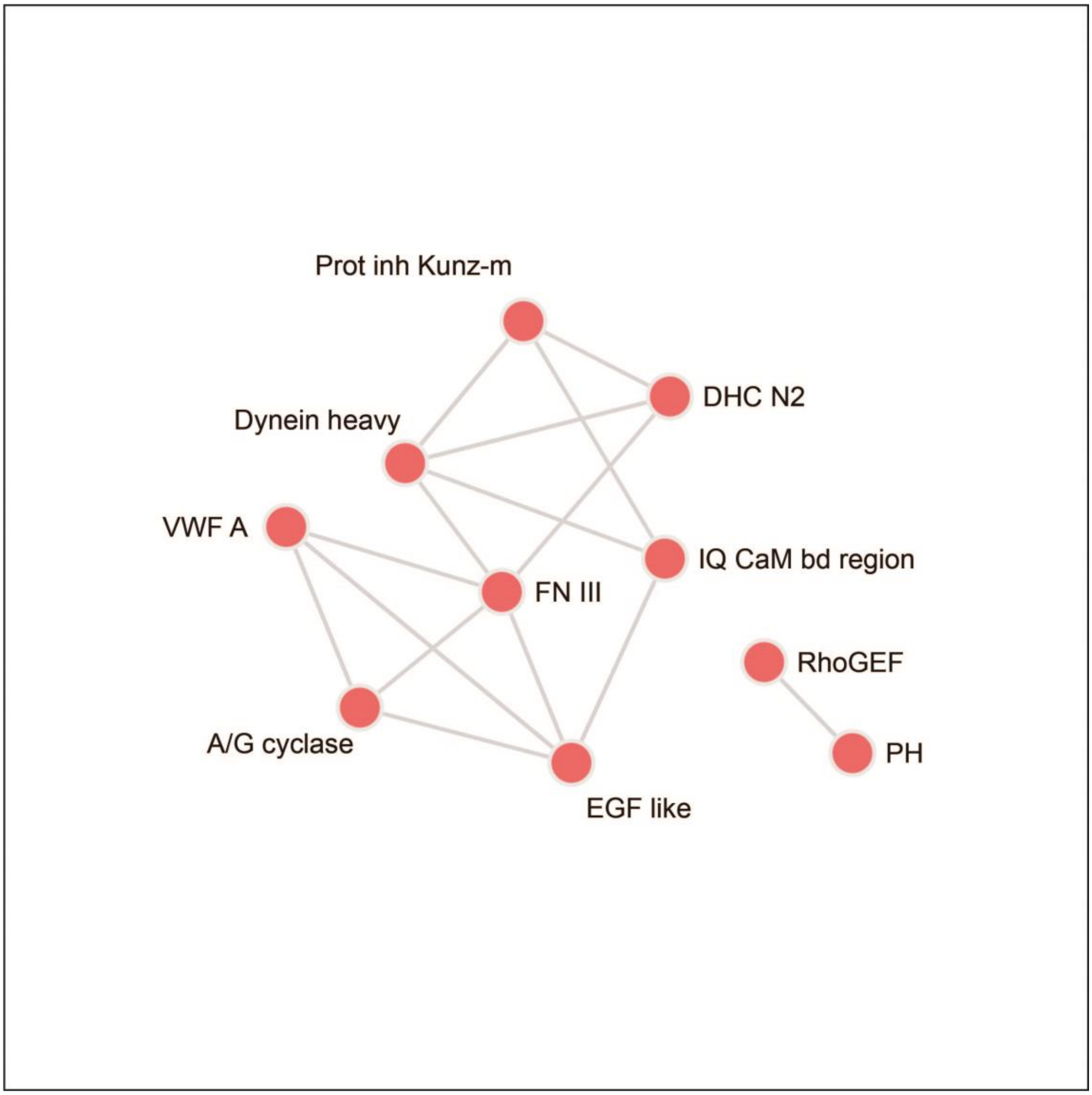

Figure S3. The functional association network constructed by Enrichr, Related to Table 2.

Table S1. Summary of sequencing data and comparison of 1196 individual samples collected in the previous period, Related to Figure 1.

	BCS samples		BCS controls	
	Mean	25th-75th quantile	Mean	25th-75th quantile
Raw Reads (All reads)	185476216	(160659952-206326932)	171490686	(153826163-183413069)
Mapped Reads	185039725	(159884660-205998269)	171087306	(153443428-183050974)
Fraction of Mapped Reads	99.76	(99.72-99.84)	99.76	(99.74-99.81)
Fraction of duplicated reads	28.10	(21.82-32.84)	28.61	(22.28-33.96)
Target Reads	132820278	(113562700-149155994)	139119912	(124430916-147809283)
Fraction of Target Reads	71.87	(70.71-75.63)	81.07	(79.65-82.49)
Average depth(rmdup)	129.44	(108.84-145.92)	134.93	(120.59-147.31)
Coverage in targeted region (>=30x)	95.45	(94.69-96.57)	95.34	(94.58-96.2)
Average depth in flanking region	47.55	(40.05-53.9)	43.87	(38.3-47.44)
Coverage in flanking region (>=30x)	48.20	(42.62-53.21)	42.56	(38.13-45.90)

Table S2. Comparison of age and gender between cases and controls in the BCS cohort, Related to Figure1.

	Group	Number of samples	Average age	P value	Statistical method	Gender		P value	Statistical method
						Male	Female		
Before sample filtering *	BCS samples	500	45.43	1.66e-45	Student t test	212	288	0.02	Fisher exact test
	BCS controls	696	35.35			247	446		
After sample filtering	BCS samples	483	45.59	1.23e-46	Student t test	207	276	0.01	Fisher exact test
	BCS controls	688	35.33			244	444		

* In the data set before filtering, there are three samples in the BCS control group without clear gender information.

Table S4. The list of known genes associated with vascular malformation, Related to Figure 4.

**Gene symbol**			
MAP3K3	*ENG*	*NAGA*	*VEGFB*
ET	*EPHB4*	*NEMO*	*VEGFR3*
BMP9	*EPO*	*NOTCH1*	*VEGFR2*
MADH4	*F5*	*PCNA*	*VEGFC*
ALK1	*FAM111B*	*PDCD10*	*VG5Q*
ACE	*FLT1*	*PIEZO1*	*RNF213*
AKT1	*FLT4*	*PIK3CA*	*NOTCH3*
ACTA2	*FLVCR2*	*PIK3R2*	*HTRA1*
ACVRL1	*FOXF1*	*PON1*	*VEGF*
ANGPT1	*FOXC2*	*PTEN*	*TGFBR1*
ANTXR1	*GDF2*	*RASA1*	*TGFBR2*
ARHGAP31	*GLMN*	*SLC7A14*	*ACVLR1*
ATM	*GNAQ*	*SLC2A10*	*IL1b*
ATR	*GLOMULIN*	*SMAD4*	*ITGB8*
BMPR2	*GUCY1A1*	*SNORD118*	*ANGPTL4*
CCM2	*IKBKG*	*SNRK*	
CCM1	*IGFBP7*	*SOX18*	
CCM3	*IL1RN*	*STAMBP*	
CCND2	*IL6*	*STN1*	
CLEC14A	*IDH1*	*TALDO1*	
COL4A1	*IDH2*	*TEK*	
CTC1	*JAK2*	*TIE1*	
CTD	*KDR*	*TIE2*	
DLL4	*KRAS*	*TMEM173*	
DOCK6	*KRIT1*	*TREX1*	
DUSP5	*LBR*	*VHL*	
EIF2AK4	*MGC4607*	*VASN*	
ELMO2	*MRE11*	*VEGFA*	
